# A Missed Case of Occult Bilateral Temporomandibular Dislocation Mistaken for Dystonia

**DOI:** 10.1155/2015/753260

**Published:** 2015-09-07

**Authors:** Evelyn Lee, Jan Shoenberger, Jonathan Wagner

**Affiliations:** ^1^LAC+USC Medical Center, 1200 North State Street 1060H, Los Angeles, CA 90033, USA; ^2^LAC+USC Medical Center, Keck School of Medicine of USC, Los Angeles, CA, USA

## Abstract

A 24-year-old male with a history of psychiatric disorder and no prior significant temporomandibular joint (TMJ) pathology presented to the emergency department for “lockjaw.” Plain film X-rays of the mandible were read as unremarkable by an attending radiologist, leading to the initial diagnosis of medication-induced dystonic reaction. Following unsuccessful medical treatment a maxillofacial computed tomography (CT) was ordered. CT confirmed bilateral dislocation, illustrating the importance of clinical judgment, and limitations of certain radiographic images. The authors believe this case to be the first reported case in the medical literature of bilateral anterior TMJ dislocation with a false negative X-ray.

## 1. Case Presentation

A 24-year-old Hispanic male with a past medical history of an unknown psychiatric disorder was sent to the emergency department (ED) for “lockjaw” after opening his mouth widely while yawning. The patient was unable to close his mouth and complained of associated left jaw pain. He denied history of similar episodes, recent changes in his haloperidol dosing, history of trauma to the face, jaw clicking, dislocations, or other temporomandibular joint pathology.

On exam, the patient's mouth was symmetrically wide open with his lower mandible protruding forward. On palpation, there was mild tenderness to palpation of the left ramus and temporomandibular joint (TMJ) with no significant periauricular depressions. The patient was calm and able to communicate with mildly slurred speech. He was able to close his lips to form words and had minimal drooling, but the jaw remained open.

**Figure 1:**
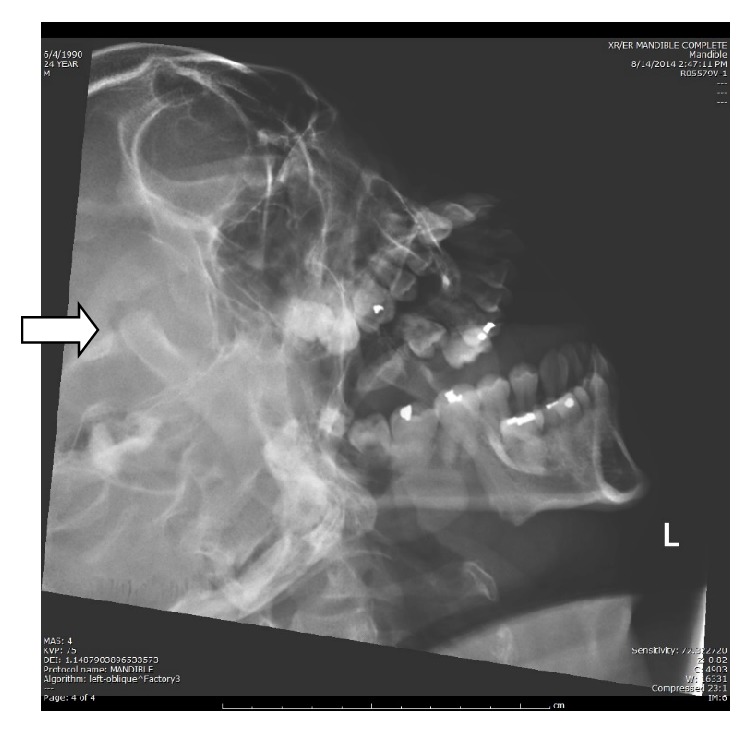
X-ray mandible series (left lateral view). The arrow is pointing to the condylar head.

Common to many EDs, panoramic X-ray capabilities were not immediately available, and, therefore, plain film X-rays of the mandible were pursued. The mandible series, which includes anteroposterior, bilateral, and submental-vertex views, was read as an “unremarkable plain film of the mandible” (Figure 1) by an attending radiologist. Upon consideration of the X-ray findings, the patient's calm demeanor, his ability to communicate relatively easily with little discomfort, and his history of psychotropic medication use, the presentation was thought to be consistent with a medication-induced dystonic reaction, rather than a dislocation. Intravenous diphenhydramine 25 mg, lorazepam 1 mg, and benztropine 1 mg were sequentially administered to the patient for presumed dystonia.

**Figure 2:**
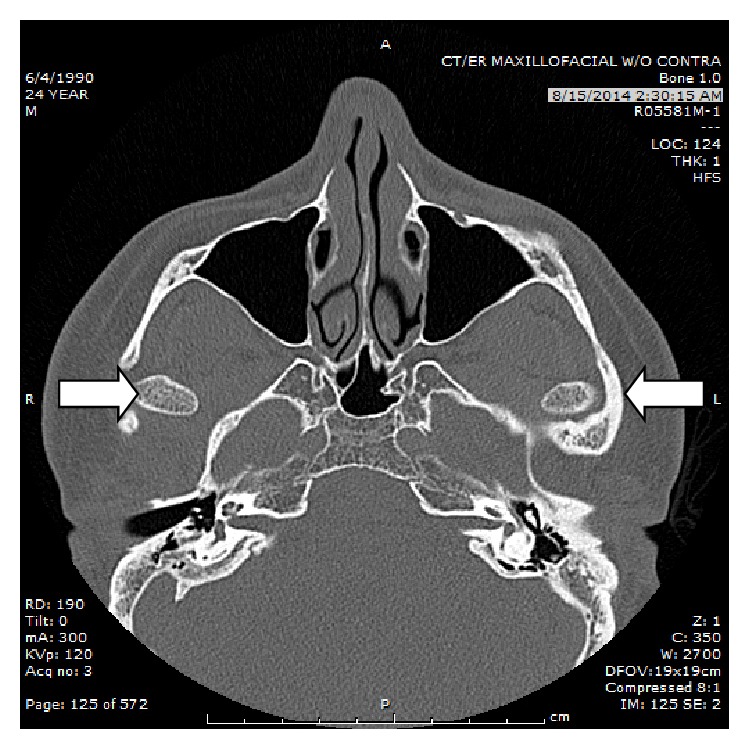
CT showing bilateral condylar heads (arrows) anterior to the glenoid fossa.

Following a period of observation, multiple reassessments, and a lack of response to the medications, the decision was made to pursue advanced imaging. Despite the normal X-ray read, a maxillofacial computed tomographic (CT) scan was ordered for further evaluation of the bony structures of the face, specifically looking for occult bilateral TMJ dislocation. The radiologist read of the CT stated that “the mandibular heads are dislocated anteriorly out of the TMJs bilaterally” (Figure 2).

The patient consented for reduction and was given midazolam 2 mg IV. Using the classic technique of placing the thumbs intraorally on the lower molars while applying steady pressure downward and posteriorly, the condylar heads were successfully reduced on the first attempt. An intraoral examination was not performed after the reduction, but the patient was able to easily open and close his jaw without difficulty, crepitus, or clicking. He also stated that his bite felt normal.

## 2. Discussion

Generally, atraumatic dislocations result from wide mouth opening (dental extraction, vomiting, laughing, oral sex, etc.) with yawning described as the most common associated mechanism [1–4]. Anterior head dislocations are most frequently seen, but the condylar heads can also slip inferiorly, medially, or posteriorly [2, 3]. Anterior TMJ dislocations occur when the mandibular condyle is displaced from the glenoid fossa anteriorly beyond the articular eminence and cannot be self-reduced [1, 5, 6]. Bilateral dislocations occur more often than unilateral dislocations [7, 8].

Typical clinical findings for mandible dislocation include the inability to close the mouth, visual or palpable depressions in the preauricular areas, prominent lower jaw or new onset prognathism, lateral chin deviation if unilaterally dislocated, excessive drooling, difficulty speaking, and a general uncomfortable appearance [1, 3, 5].

The diagnosis of atraumatic mandibular dislocation frequently requires no imaging and should be obvious clinically in the majority of cases [3, 5, 9]. The decision to image is determined by practitioner preference and/or on a case-by-case basis, as no set guidelines for imaging exist. The American Academy of Oral and Maxillofacial Radiology suggests evaluating the entire clinical picture and determining whether imaging would assist with diagnosis given the risks of radiation exposure and cost [10, 11]. The general practice in the ED is to first start with plain film X-rays. The most important views are panoramic, transpharyngeal, transcranial, and submental-vertex views with recommendations for transcranial views [11, 12].

Currently, CT imaging is used more frequently than plain radiography in both traumatic and atraumatic mandibular dislocations, as it allows for evaluation of fractures, dislocations, and osseous changes [13, 14]. Magnetic resonance imaging (MRI) is rarely needed and is used primarily for diagnosing chronic changes and internal derangement of the TMJ [12, 15].

Internal derangement of the TMJ mainly involves the relationship of the mandibular condyle and the articular disc that sits between the condyle and the glenoid fossa. The articular disc helps with smooth joint movement. When displacement or derangement of the disc occurs, pain, clicking, popping, or joint locking can occur [15, 16]. When the articular disc displaces posteriorly, the mouth is locked in an open position, otherwise known as “open lock.” The posteriorly displaced disc functions as a mechanical block that does not allow the condyle to return to the glenoid fossa and can appear clinically similar to a mandible dislocation [17]. Open lock is an uncommon finding and is rarely diagnosed in the emergency department. Therefore, if a mandibular dislocation is suspected, the appropriate next step is treatment with manual reduction [18]. In an outpatient setting, unenhanced MRIs or cone beam CTs (which use lower radiation to focus on the TMJ) are used to help evaluate and diagnose internal derangement of the TMJ. However, MRIs are the modality of choice in the evaluation of the articular disc [10, 14, 15]. Typically, treatment is nonsurgical and focused on patient education and counseling regarding behavior modification and anti-inflammatory medications. Serial muscle exercises that help to restore neuromuscular control, improve TMJ mobility, and decrease TMJ stress are recommended and may be useful in the treatment and prevention of future episodes of open lock [16].

In this case, the patient appeared comfortable and was speaking relatively clearly. He also was without major pain or anxiety. Due to the incongruence between the patient's clinical appearance and the described typical presentation of an acute bilateral mandible dislocation, in conjunction with the radiologist read of a negative mandible series, the coincidental nature of the patient's history of yawning with symptom onset was falsely assumed.

Although the final diagnosis of bilateral mandibular dislocation was slightly delayed from the initial X-rays to the final CT findings, the medications used to treat the suspected dystonic reaction likely aided in muscle relaxation and a simple and successful reduction in the emergency department.

## 3. Conclusion

While plain films of the mandible can provide a great deal of information, they may also mask the true underlying disorder [19]. Ultimately, it is important to keep an open mind when considering the differential diagnosis when the patient presentation does not correlate to the expected radiologic results. It is important to realize the limitations of certain radiographic images and the importance of clinical judgment. The authors believe that this is the first case of bilateral anterior TMJ dislocation with a false negative X-ray reported in the medical literature to date.
